# High-resolution multimodal photoacoustic microscopy and optical coherence tomography image-guided laser induced branch retinal vein occlusion in living rabbits

**DOI:** 10.1038/s41598-019-47062-2

**Published:** 2019-07-22

**Authors:** Van Phuc Nguyen, Yanxiu Li, Wei Zhang, Xueding Wang, Yannis M. Paulus

**Affiliations:** 1 Department of Ophthalmology and Visual Sciences, University of Michigan, Ann Arbor, MI 48105 USA; 2 Department of Biomedical Engineering, University of Michigan, Ann Arbor, MI 48105 USA; 3 NTT-Hi Tech Institute, Nguyen Tat Thanh University, Ho Chi Minh, Vietnam; 4 Department of Ophthalmology, Xiangya Hospital, Central South University, NO. 87 Xiangya Road, Kaifu District, Changsha, Hunan 410008 PR China

**Keywords:** Biophotonics, Photoacoustics, Experimental models of disease

## Abstract

Joint high-resolution multimodal photoacoustic microscopy (PAM) and optical coherence tomography (OCT) was developed to improve the efficiency for visualizing newly developed retinal neovascularization (RNV) and to monitor the dynamic changes of retinal vein occlusion (RVO) in living rabbits. The RNV and RVO models were created in New Zealand rabbits by Rose Bengal laser-induced RVO. Dual modalities imaging equipment, including color fundus photography, fluorescein angiography (FA), OCT, and PAM, was used to image and assess the changes of retinal vasculature. *In vivo* experimental results exhibited that not only the treatment boundaries and the position of the occluded vasculature but also the structure of individual RNV were markedly observed using PAM platform with great resolution and high image contrast. The laser light energy of 80 nJ was used to induce photoacoustic signal, which is approximately half the energy of the American National Standards Institute safety limit. A cross-sectional structure of RNV was identified with the OCT modality. Furthermore, vibrant transformations in the RNV and the retinal morphology were examined at different times after laser occlusion: days 4, 28, 35, 49, and 90. PAM revealed high contrast and high resolution vascular imaging of the retina and choroid with amplified penetration depth. Through the present custom-built imaging system, both RNV and RVO can be reconstructed and observed in two and three dimensions. A unique dual modality A unique dual modality PAM and OCT can help precisely visualize and distinguish individual microvessels, microvessel depth, and the surrounding anatomy. Thus, the proposed multimodal ocular imaging platform may offer a potential equipment to enhance classification of microvasculature in a reliable and proficient manner in larger rabbit eyes.

## Introduction

Retinal vein occlusion (RVO) commonly produces vision loss and impaired vision in older patients^1^ and is the second most common retinal vascular disease, following diabetic retinopathy^2^. RVO impacts more than 16 million people in the United States and occurs in 1–2% in individuals older than 40 years old^3^. The prevalence of RVO increases with age and is expected to increase with the aging population^4^. Half of RVO occurs in individuals older than 65 years old^5^. The two major anatomic forms of RVO include central and branch retinal vein occlusion^6^. Hayreh *et al*. have reported that RVO can be divided into two sub-groups: non-ischemic and ischemic^2^. 20% of eyes with ischemic RVO develop retinal neovascularization (RNV)^1,7^. RNV frequently develops at the margin between nonperfused (ischemic) and perfused retina^8^. Therefore, it is critical to developing an imaging technique to monitor and to understand RVO pathogenesis.

Recently, animal models have been useful to test new treatments for cancer, ischemia, diabetes, and branch and central retinal vein occlusions^9–12^ due to their relative low cost and availability. For studies involving eye diseases, rabbits have often been used because the rabbit eye axial length of 18 mm is similar to the human eye axial length of ~23 mm. However, rabbit eyes have some notable differences from human eyes. Rabbits do not have a fovea and the retinal vasculature is merangiotic in rabbits and holangiotic in humans. Rabbits also have a horizontal streak of myelinated retinal nerve fiber layer (MRNFL) called the medullary ray that does not exist in humans. The MRNFL appears as a grey-white, sharply demarcated patch with frayed or feathered margins along the retinal nerve fiber layer that is contiguous with the optic nerve^13–15^. In humans, MRNFL occurs in 0.57 to 1% of the population and may affect vision^16^. While animal models have their utility, in some cases animal models may not share the same pathophysiology or response to treatment as the human diseases they seek to mimic. Several investigations on retinal vein occlusion have used rabbits as an animal model.

Several ocular imaging modalities have been investigated and developed to evaluate RVO, retinal capillary nonperfusion, and RNV including fluorescein angiography (FA) and indocyanine green angiography (ICGA)^17–19^. However, FA only visualizes the superficial capillary plexus^17^. In addition, FA can only detect about 50% to 60% retinal capillary nonperfusion among RVO cases, making it difficult to distinguishing between ischemic and nonischemic RVO^8^. ICGA is used to detect neovascular layers underneath the retinal pigment epithelium (RPE) and through media opacities, including hemorrhage and cataract. Both ICGA and FA cannot specify the depth of the vasculature, invasive technique and can cause several side effects such as nausea, vomiting in up to 10% of patients, and in rare instances anaphylaxis which can cause death^20^. Recently, several optical ocular imaging platforms including optical coherence tomography (OCT), OCT angiography (OCTA), scanning laser ophthalmoscopy (SLO)^17,21^, and photoacoustic microscopy (PAM) have been widely investigated for visualizing RVO. Each modality has its own strengths and drawbacks when evaluating retinal ischemia *in vivo*. Although OCT provides an opportunity to non-invasively visualize superficial retinal vascular disease with a high resolution, it struggles to visualize the choroid and choriocapillaris vasculature. OCT provides limited information on these important layers outside of thickness measurements^22^. SLO can achieve visualization of the smallest retinal capillaries, but SLO has limited image contrast and depth resolution, resulting in the inability to differentiate between vascular beds^23,24^. OCTA offers both structural and vascular information, which permits assessment of vasculature with depth information; however, it cannot detect leakage, is limited in its ability to visualize microaneurysms, and is unable to visualize the choroid and choriocapillaris vasculature^25,26^.

Photoacoustic (PA) imaging has been developed and used as an emerging, non-invasive and non-ionizing imaging technique to evaluate various types of ocular tissues. Compared to other imaging modalities, PA ocular imaging can identify the objects with high sensitivity, high-contrast, high-resolution, and high depth of penetration^9,27–31^. de la Zerda *et al*. demonstrated that retinal vessels could be detected using photoacoustic ocular imaging^27^. Recently, several investigators have introduced a combined OCT with PAM imaging system to advance examine retinal vasculature, RPE, and microvasculature^29,32,33^. Previously, our group has reported that an integrated PAM and OCT imaging system could be applied for label identifying retinal and choroidal vessels^11,34^ in living rabbits with great contrast and excellent resolution^11,35^. Our custom-made dual imaging equipment achieves a lateral resolution of 4.1 µm for PAM and 3.8 µm for OCT respectively, which allows researchers visualize and distinguish between retinal and choroidal vasculatures. Therefore, integrated PAM and OCT platform can possibly be used to further differentiate RNV and RVO.

The focus of the current study is to investigate a multimodal imaging modality for charactering laser-induced retinal vein occlusions using color fundus photography, FA, PAM, and OCT. RVO and RNV were created using intravenous administration of dye-enhanced photocoagulation Rose Bengal followed by high power laser illumination. Multimodal PAM and OCT were used to monitor the location of RVO and monitor neovascularization. In addition, the acquired PAM images of the treated retinal vasculatures were used to evaluate the changes in the retinal morphology and the spatial degree of retinal vascular changes.

## Results

### *In vivo* PAM and OCT

**Figure 1:**
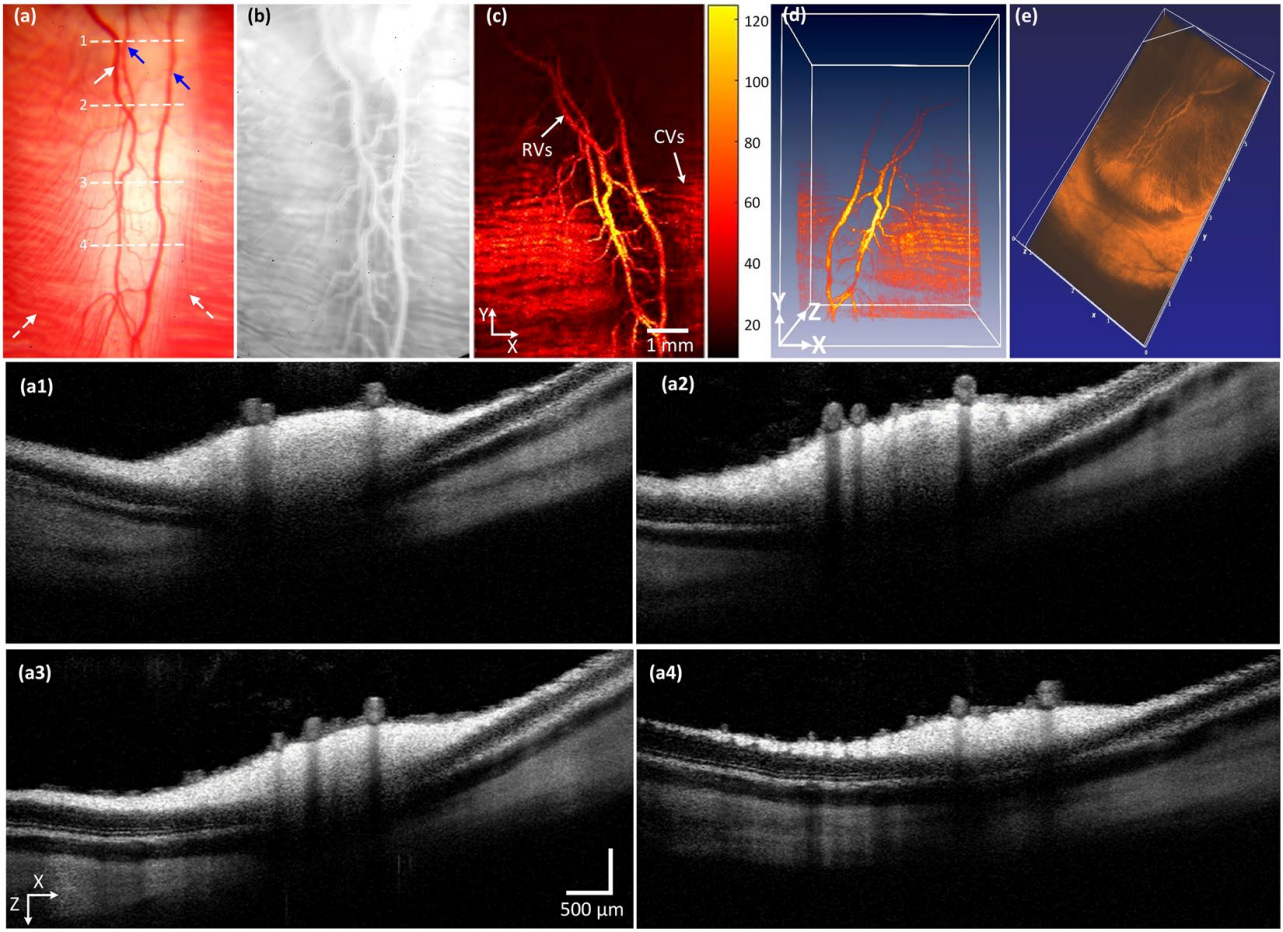
Multimodal imaging of retinal blood vessels in rabbits: (**a**) color fundus photography of retina. Blue arrows represent the position of arteries, white arrow indicates the position of the vein and white dotted arrows show the location of choroidal vessels. White dotted lines exhibit the selected scanning area. (**b**) Fluorescein angiography image showing retinal, choroidal and capillaries. (**c**) Corresponding maximum amplitude projection (MAP) PAM images before retinal vein occlusion. The MAP image illustrates clearly structure and high contrast of retinal vessels. (**d**,**e**) 3D volumetric PAM and OCT image, respectively. (**a1–a4**) Cross-sectional OCT images acquired along the scanning lines from Figure (**a**). The OCT image showing CVs, RVs, and retinal layers.

To evaluate for changes in retinal vasculatures post-photocoagulation, the rabbits’ retinal vasculatures were visualized *in vivo* using integrated PAM with OCT imaging. The acquired maximum intensity projection (MIP) PAM images of the retinal vascular were achieved at 580 nm since oxygenated hemoglobin has strong absorption optical light at that wavelength. Figure 1 shows a baseline image of major retinal vessels prior laser photocoagulation induced RVO. A digital color fundus image of the rabbit retina before acquiring baseline OCT and PAM imaging, and laser-induced RVO is illustrated in Fig. 1(a). The fundus image displays the morphology of the choroidal vessels (white dotted arrows), retinal arteries (blue arrows), and veins (white arrows). The retinal arteries and veins were differentiated by evaluating sequential FA images, particularly the early transit phase images, as shown in Fig. S1. Figure 1(b) shows an FA image. The morphology of both artery and vein is visualized. FA images were used to evaluate blood flow before and after RVO. Figure 1(c) illustrates the corresponding PAM image of the retinal vascular acquired along the selected region of interest (ROI) presented in Fig. 1(a). The acquired PAM image presents the individual morphology of the retinal vascular with high contrast. Furthermore, the three-dimensional (3D) volumetric visualization of the acquired data was rendered and illustrated in Fig. 1(d). The main retinal vascular such as retinal (arteries and veins), choroidal, and microvessels were clearly identified and visualized, revealing the ability of photoacoustic modality for recognition of individual retinal vessels *in vivo*. Figure 1(a1–a4) present the OCT images obtained along the dotted lines 1 to 4 displayed on Fig. 1(a). These OCT images were captured at different positions along the retinal vessels and reveal intact retinal layers. Figure 1(e) exhibits 3D OCT visualization of the morphology of the retinal vasculature. Similar to the 3D PAM images, the OCT volumetric image also clearly illustrates the retinal branching vascular network.

**Figure 2:**
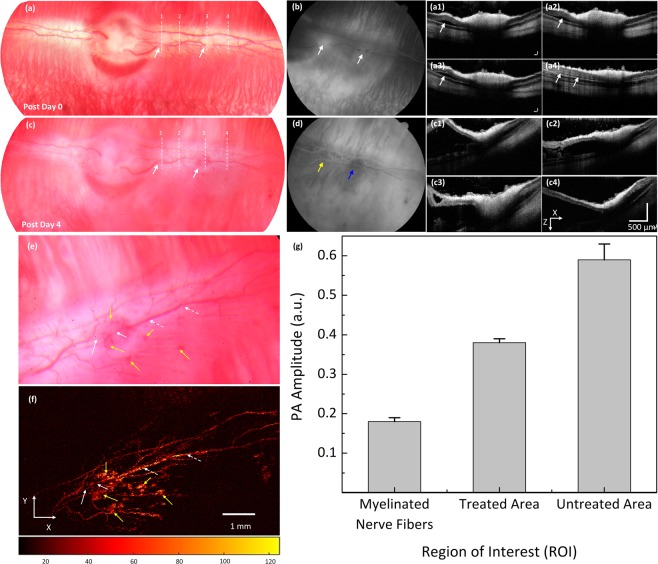
*In vivo* analysis of a retinal vein occlusion. (**a**) Color fundus image of retinal acquired at 15 min after laser application. White arrows indicate the position of the treated area, whereas white dotted lines show the selected scanning region. (**b**) FA image showing the occluded vessels. White arrows exhibit the position of interruption of blood flow, indicating vessels completely occluded. (**a1–a4**) Corresponding OCT images acquired along the dotted lines as shown in Figure (**a**). The OCT showing retinal slightly detached after photocoagulation. (**c**) Color fundus image acquired on day 4 after treatment. White arrows show hemorrhages. (**d**) FA shows the occluded vessels at the second treatment area while the first treatment area (near the optic nerve) reperfusion. (**c1–c4**) Post-occlusion B-scan OCT images acquired at white dotted lines in Figure (**c**). The OCT image showing significant retinal detachment. (**e**) Magnification image of the retinal vessels extracted from Figure (**c**). (**f**) Corresponding MAP PAM images. White arrows show the position of occluded vessels. White dotted arrows depict the location of untreated vessels. Yellow arrows exhibit the region of hemorrhage. (**g**) Comparison of PA amplitudes at different region of interests (ROI) (*p < 0.001 and N = 4).

After acquiring baseline images, laser-induced retinal vein occlusion was performed on the rabbit retinal vessels of interest, and the specific location for vascular occlusion were selected and illuminated with high power laser light. Then, the rabbit RVO model was evaluated by multimodal imaging equipment as shown in Fig. 2. The retinal vessels after laser-induced RVO were first examined by the color fundus photograph system (Fig. 2(a)). The position of laser treatment is indicated by the white arrows. The color intensity of the occluded vessels on the fundus photograph was significantly changed, which implied decreased intravascular blood volume. The FA image Fig. 2(b)) demonstrated the treated vessels were completely occluded. The blood flow was monitored from the sequential FA images (Fig. S2). As shown in Fig. S2, the occluded arteries and veins require about 20 s to fill completely. Figure 2(a1–a4) show the B-scan OCT image obtained from the dotted lines 1–4 depicted in Fig. 2(a). The white arrows indicate the deposit of subretinal fluid causing a localized serous retinal detachment in the first 15 min following laser irradiation that peaked at 4 days after treatment and resolved by 7 days. Other changes in the occluded vessels observed on day 4 after laser irradiation included blood vessel non-perfusion, reperfusion, and hemorrhage. There was a hemorrhage observed on the retinal vessels in the treated areas (Fig. 2(c)). On FA (Fig. 2(d)), the treated vessels were totally blocked (blue arrow) or partially blocked (yellow arrow). There was delayed filling of retinal vessels by 4–5 s in comparison with normal vessels at the treated area near the optic disc. In contrast, no blood flow was detected at the second treated area as indicated by the blue arrow. Figure 2(c1–c4) show the cross-sectional OCT images acquired along the dotted lines 1–4 from Fig. 2(c). These B-scan OCT images exhibit the variation in the retinal layers. Subretinal fluid persisted at day 4 after laser irradiation, and the serous retinal detachment increasingly improved and entirely revolved by day 7 post-treatment.

To visualize the margin of the treated vessels after laser-induced RVO, PAM was performed. Figure 2(e) depicts a color fundus photograph extracted from Fig. 2(c). Figure 2(f) shows the corresponding PAM images of the treated vasculatures presented in Fig. 2(e). The photoacoustic image highlights the pathologic changes in the retinal vasculatures after treatment. At the laser irradiation site (white arrows), the treated vessel was blurred and achieved a weaker contrast when compared to the untreated area (white dotted arrows). The detected position of hemorrhage at the treated area and at the capillaries are marked by yellow arrows with high image contrast. The retinal hemorrhage was gradually decreased and completely disappeared by day 28 (Fig. S3). Figure 2(g) shows the measured photoacoustic amplitudes at various regions of interest (i.e., untreated retinal vessel, treated retinal vessel, and myelinated nerve fibers) in the acquired PAM image from Fig. 2(f) to evaluate the dynamic optical absorption behavior of retinal vessels after photothrombosis. The computed photoacoustic contrast (PA_untreated_/PA_treated_ - 1) × 100) within treated retinal vessels decreased and was 55% lower than that of untreated retinal vessels. In comparison with adjacent tissues, the calculated photoacoustic image contrast of the treated and untreated vessels was about 1.11 and 2.28 times higher than that of PA amplitude from the myelinated nerve fibers (i.e., PA amplitudes = 0.38 ± 0.01 (a.u.) and 0.59 ± 0.04 (a.u.) for treated and untreated retinal vessels vs. 0.18 ± 0.01(a.u.) for myelinated nerve fibers, p < 0.002).

**Figure 3:**
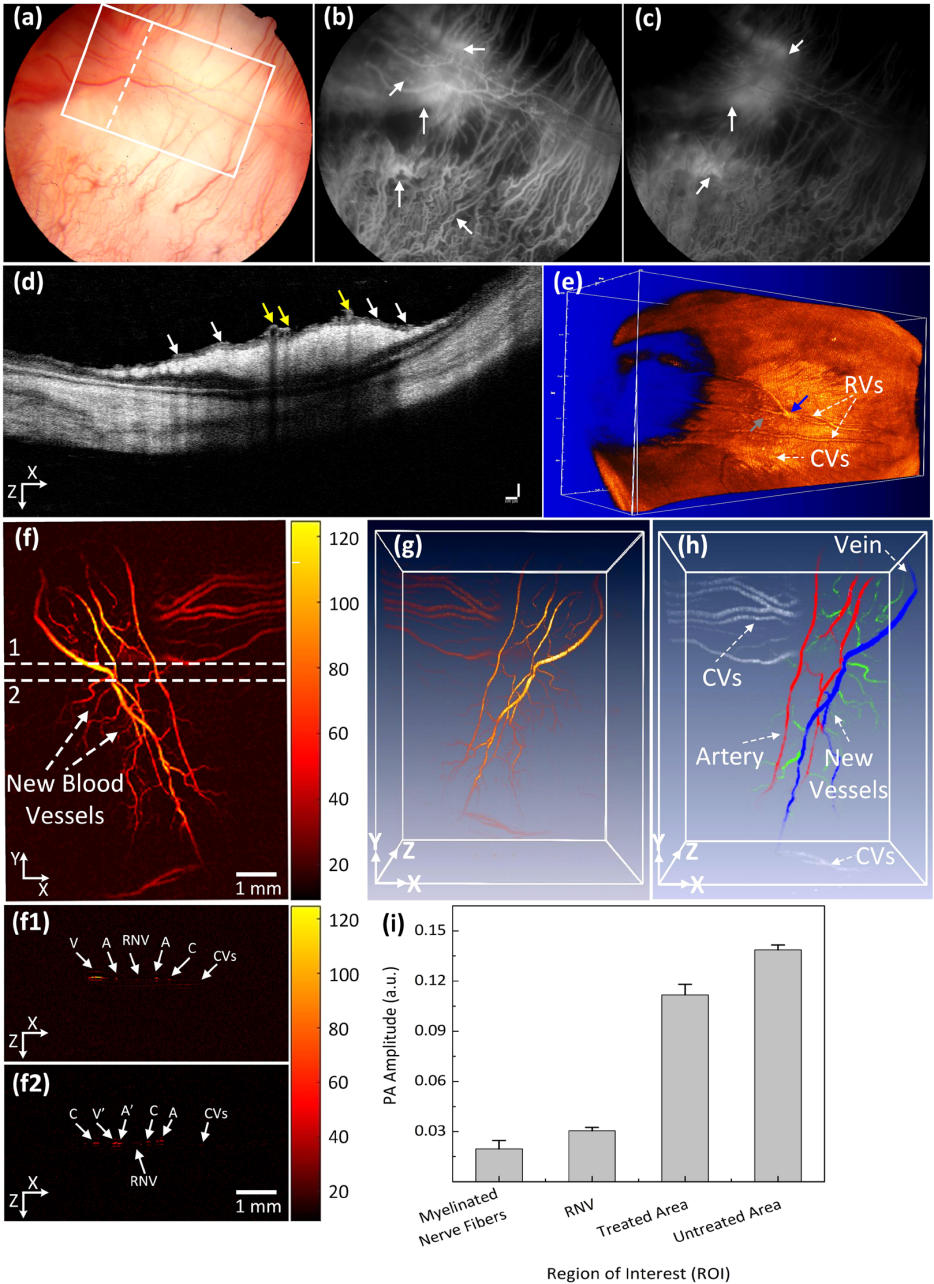
Multimodal FA, OCT, and PAM imaging of retinal neovascularization. (**a**) color fundus image of the retina. White rectangle shows the selected scanning region (PAM) and whited dotted line show the scanning line position (OCT). (**b**,**c**) FA image at early phase and late phase. White arrows show the position of retinal neovascularization. (**d**) B-can OCT image acquired along the dotted line in Figure (**a**). White arrows indicate the position of RNV, whereas yellow arrows represent the location of treated retinal vessels. (**e**) 3D rendering OCT image. Blue arrow shows the treated areas with higher contrast in comparison with the background. Gray arrow depicts the location of the artery. (**f**) Corresponding MAP PAM images after laser irradiation at day 28. The MAP PAM images show clearly the structure of individual retinal blood vessels including RNVs, CVs, RVs, and capillaries. The PAM image after laser treatment shows clearly the change of treated vessels. (**g**) 3D rendering PAM images (see Visualization 2). (**h**) Segmentation image of retinal vessels for RNV identification. Pseudo-color blue, red, white, and green indicate the position of vein, artery, choroidal vessels and RNV, respectively. (**f1** and **f2**) B-scan image from upper and lower dotted lines in (**f**). (**h**) Comparison of maximum PA signals at different positions on rabbit retinal vessel after treatment (*p < 0.001 and N = 4). The PA signal slightly decreased after laser treatment from 0.14 ± 0.003 (a.u.) to 0.11 ± 0.006 (a.u.).

At day 28 post laser irradiation, all the occluded retinal vessels had revascularized. Nevertheless, the vascular outline remained quite irregular with increased vascular tortuosity in both treated and untreated vessels (Fig. S4). In addition, tortuosity increased in choroidal vessels, which is consistent with a previous report by Cohefski *et al*.^36^. Their studies have shown that shunting of retinal vessels occurs for drainage by neighboring veins. The tortuosity was determined by using the relative length variation method^37^. The choroidal vessels at the laser site were also significantly changed and reduced as shown in Fig. 3(a). In addition, RNV developed and was visualized on FA (Fig. 3(b,c)). The white arrows indicate the location of RNV on both early (Fig. 3(b)) and late (Fig. 3(c)) phase FA images. Most RNV occurs near the optic disc. Figure 3(d) exhibits the OCT images obtained from the selected scanning line shown in Fig. 3(a). The yellow arrows highlight the native retinal vasculature whereas the white arrows indicate the position of the newly developed RNV. Additionally, no retinal detachment was observed on the OCT image. Figure 3(e) depicts a 3D OCT volumetric image, showing the structural features of the vascular networks of the rabbit retina. The revascularized vessels, the characteristics of neovascularization, and the retinal blood vessels are highlighted by the blue, gray and white arrows, respectively. This demonstrates that using OCT, a clinician is able to monitor the development of RNV.

The potential use of PAM for visualizing RNV was examined using the rabbit models. The RNV was fully imaged and subsequently rendered in 2D and 3D.achieved Fig. 3(f) presents the maximum amplitude projection (MAP) of the PAM images of the retinal vessels achieved at the scanned area (white rectangle) displayed in Fig. 3(a). Unlike FA, the PAM image was captured without the injection of any contrast agent. Figure 3(f) demonstrates PAM image of the rabbit RNV with high contrast and high-resolution. Figure 3(f1,f2) B-scan PAM images captured along the upper and lower dotted lines in Fig. 3(f) respectively are the B-scan PAM images captured along the upper and lower dotted lines in Fig. 3(f) respectively to compare the axial penetration depth of the treated retinal vessels, RNV, and untreated vessels. The untreated retinal arteries and veins (marked as A and V) were clearly visualized on the B-scan PAM images, whereas the cross-sectional images of the treated arteries and veins were blurred and reduced the PA image contrast (marked as A’ and V’). The depth information of capillaries (denoted as C), RNV, and choroidal vessels (denoted as CVs) was unchanged. It was noted that the vessel diameter of the untreated veins was (on average?) 132% higher than that of the treated veins (vessel diameter = 121.88 ± 7.18 µm for untreated veins vs. 91.88 ± 12.81 µm for treated veins; p < 0.05), indicating that the treated veins were thinner. Figure 3(g) is a 3D volumetric rendering of retinal vessels. The retinal vascular network was more effectively identified, and the morphology of distinct vessels was easily differentiated. Despite the high image contrast, the extensive, closely knit branching network of the retinal micro-vessels makes the detection of RNV quite difficult. Therefore, a vessel segmentation algorithm was applied to identify individual retinal vasculature and to trace the changes in vessel size. The boundaries of the arteries, veins, RNVs, and choroidal vessels (CVs) were detected and distinctly segmented as displayed in Fig. 3(h). For visualization, the regions of the retinal vessels were highlighted with different pseudo-colors (red, green, blue, and white). Red indicates the location of arteries, green indicates RNV, blue indicates the position of veins, and white indicates CV. RNV was identified within each image, demonstrating the potential of PAM for detection and evaluation of RNV. Figure 3(i) shows the comparison of PA amplitudes from four different regions of interest (ROI) including RNV, treated, untreated retinal vessels, and myelinated nerve fibers from Fig. 3(f). The PAM image contrast of the RNV, treated, and untreated retinal vessels were approximately 1.5, 5.5, and 7.0-fold higher than the signal from the myelinated nerve fibers (PA amplitudes = 0.14 ± 0.003 (a.u.) for untreated vessels, 0.11 ± 0.006 (a.u.) for treated vessels, 0.03 ± 0.002 (a.u.) for RNV vs. 0.02 ± 0.005 (a.u.) for myelinated nerve fibers), respectively. Importantly, the PA image contrast of treated veins compared to untreated veins was estimated to be 0.27 ± 0.06. The treated veins induced 27% lower photoacoustic amplitudes at 580 nm, compared to the untreated site. This decrease in the photoacoustic image contrast could be utilized to classify between vessels and isolate treated vessels.

**Figure 4:**
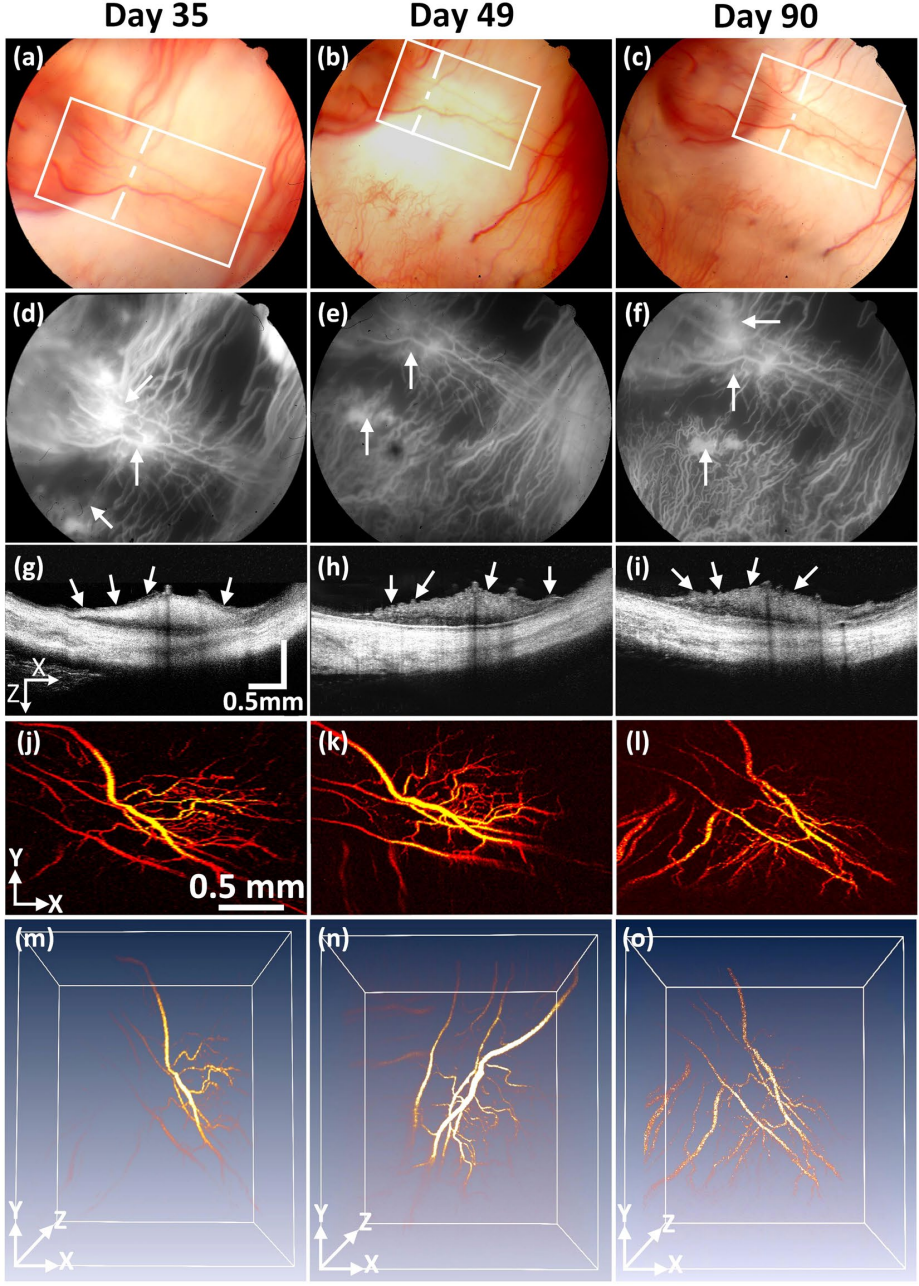
Stabilization of retinal neovascularization. (**a**–**c**) Color fundus images of the retina acquired at different days: 35, 49, and 90. (**d**–**f**) FA images. White arrows depict the position of retinal neovascularization. The retinal neovascularization was developed at day 28 after laser treatment and stabilized up to day 90. (**g**–**i**) Cross-sectional B-can OCT images acquired along the dotted lines in Figure (**a**–**c**). (**j**–**l**) Corresponding MAP PAM images acquired along the white rectangles from Figure (**a**–**c**). The PAM images illustrate the structure of individual RNVs unchange. (**m**–**o**) 3D reconstruction PAM images.

**Figure 5:**
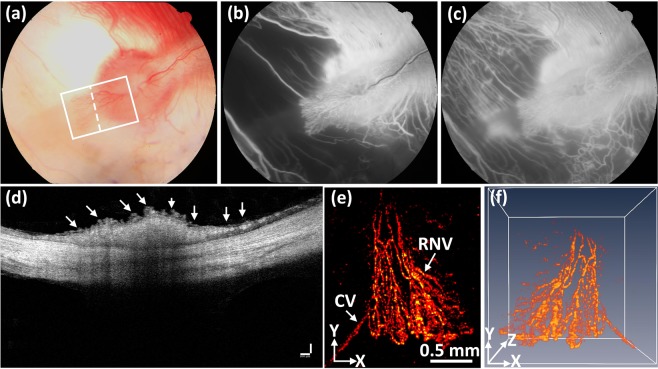
Detection of retinal neovascularization. (**a**) Color fundus image showing structure and morphology of retinal neovascularization after laser-induced RVO at day 28. White rectangle and whited dotted line indicate the selected areas. (**b**,**c**) Show the FA images at various times. (**d**) b-scan OCT images. White arrows show the location of RNV. (**e**) Corresponding PAM imaging of retinal neovascularization. The PAM images show clearly the structure of individual retinal neovascularization. (**f**) 3D rendering PAM images (see Visualization 2).

To assess the dynamic changes of the RNV model, the rabbits were monitored and followed up for 90 days after treatment. Figure 4 illustrates the RNV images various techniques obtained from several imaging techniques such as color fundus photography, FA, OCT, and PAM at various times: day 35, 49, and 90 post treatment. Color fundus photographs (Fig. 4(a–c)) allowed for visualization of retinal vessel morphology. The FA images (Fig. 4(d–f)) were co-registered with the fundus images and demonstrated RNV and leakage. As shown in these images, the morphology and the density of the RNV (white arrows) was stable from days 35 to 90 (i.e., vessel density = 41.19 ± 5.84%, 38.96 ± 3.71%, and 39.59 ± 3.24% for RNV at day 35, 49, and 90, respectively). In addition to the color fundus photographs and FA, high-resolution OCT and PAM images were acquired. Figure 4(g–i) show the two dimension OCT images obtained from the dotted lines in Figs (a–c). The white arrows denote the positions of RNV. Figure 5(j–l) represent the PAM image of the RNV acquired from the selected areas (white rectangles) shown in Fig. 4(a–c). The top view PAM images easily distinguished the boundary of the RNV, and the density of the RNV is similar over time, indicating the stabilization of the RNV. In addition, the location and margin of RNV was clearly apparent and distinct from major retinal vessels (arteries and veins) in 3D with high contrast (Fig. 4(m–o)).

In another case, the retinal vessels were completely eradicated 4 weeks after laser treatment. Then, the formation of RNV was formed and evaluated using multimodal PAM and OCT. The RNV was captured using color fundus photography at week 4, Fig. 5(a). This image was captured prior to *in vivo* PAM and OCT imaging. The selected PAM scanning area is marked by the white rectangle, while the white dotted line indicates the cross-sectional scanned line for OCT. The morphology of RNV, choroidal, and retinal vessels was observed on the FA image (Fig. 5(b,c)). It was noted that the choroidal vessels were significantly decreased at the laser irradiation site. In 100% of cases, the CVs were reduced after laser irradiation. RNV formed and increased over time. Figure 5(d) shows the OCT image captured from the dotted line in Fig. 5(a). As shown in this image, the RNV network is observed with OCT (white arrows). Figure 5(e) depicts the corresponding PAM image of the RNV displayed in Fig. 5(a). Figure 5(e) exhibits that the RNV vasculature networks and CVs are markedly recognized with a PA image contrast ((PA_RNV/CVs_/PA_Background_ - 1) × 100) of 6.00 ± 0.46, and 1.00 ± 0.12 (PA amplitudes = 0.14 ± 0.007 (a.u.) for RNV, and 0.04 ± 0.002 (a.u.) for CVs vs. 0.02 ± 0.001 (a.u.) for background), respectively. The PA image contrast of the RNV with respect to CVs ((PA_RNV_/PA_CVs_ - 1) × 100) is 2.50 ± 0.22. To better distinguish the RNV vasculature and CVs, a 3D image reconstruction was performed, Fig. 5(f). The margins and positions of RNV, capillaries, and choroidal vessels were clearly defined and visualized. Based upon the 3D image, these vessels were located in different layers (see Visualization 2). Additionally, the (average?) RNV vessel diameter was estimated to be 29.83 ± 4.17 µm, which was 97.77% thinner than choroidal vessels (59.00 ± 11.47 µm), p < 0.05. The *in vivo* PAM experiment demonstrated that PAM could serve as a potential technique to observe both the morphology of vasculature and neovascularization with high sensitivity, specificity, and spatial resolution allowing clinicians to discern individual vessels. The PAM volumetric data allowed the RVO and RNV to be precisely identified. Contrarily, previous studies using FA to monitor RVO could not visualize RVO in three dimensions^38,39^.

**Figure 6:**
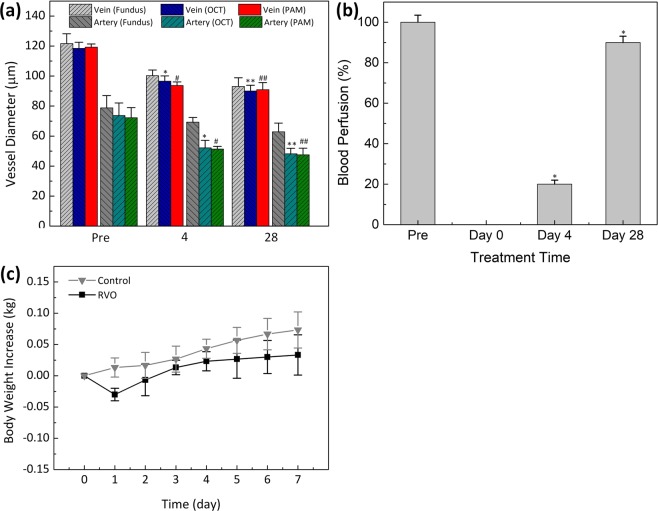
Quantitative analysis of retinal vessels after retinal vein occlusion. (**a**) Comparison vessel diameter measured from different methods: fundus, OCT and PAM (*p < 0.001 and N = 4). Both vein and artery diameters are slightly decreased after photothrobotic. (**b**) Blood perfusion as a function of treatment times (pre, day 0, day 4, and day 28) (*p < 0.001 and N = 4). (**c**) Body weight increase as a function of time after different treatment groups: control, and RVO model. The body weight was monitored every day over a period of 7 days after treatment.

All of the color fundus photography, PAM, and OCT images were used to quantitatively estimate changes in the vascular diameter as a result of laser irradiation at various times over 4 weeks (Fig. 6(a)). Each technique yielded similar results. PAM validated that the vein and artery diameters decreased from roughly 119.27 ± 2.14 and 72.25 ± 6.75 µm (pre-occlusion) to 93.75 ± 2.33 and 51.37 ± 1.77 µm (day 4 post-occlusion) and 90.95 ± 4.76 to 47.52 ± 4.46 µm (day 28 post-occlusion) respectively. Using OCT, the retinal vein and artery diameters were estimated to be 118 ± 4.03 and 73.76 ± 8.29 µm respectively, pre-occlusion. Four days post-occlusion, both decreased to 96.55 ± 3.54 and 52.18 ± 5.00 µm, respectively. Finally, 28 days post occlusion the retinal vein and artery diameters were estimated to be 90.05 ± 3.80 and 48.14 ± 3.75 µm, respectively (p < 0.01). Similarly, the measured vessel diameter was not significantly different when using fundus photography. Using fundus photography, the vein diameter was estimated to be 121.66 ± 6.55 µm (pre-occlusion) (p < 0.003), which was 2.00%, and 2.68% greater than the PAM and the OCT quantities, respectively. Color fundus photography consistently overestimated vessel size for both artery and veins.

As a result of the photothermal effect, the blood perfusion in the retinal vessels was significantly reduced and gradually plateaued, as illustrated in Fig. 6(b). Immediately after laser irradiation, the blood perfusion in the retinal vessels significantly decreased from 100% (pre-occlusion) to 0% (post-occlusion). Then, the blood perfusion gradually increases to approximately 20% at day 4 and 90% at day 28 (p < 0.05). These results demonstrated that occlusion had been successfully created after laser irradiation and then the vessels re-perfused over time. These results similarly demonstrate that the size of the treated vessel was smaller compared to the vessels pre-treatment.

To quantify the side effects and possible toxicity of the Rose Bengal application, all treated animals had their body weight recorded daily^40^. A decrease in weight is an indicator of treatment-induced toxicity in animals. Figure 6(c) highlights that the body weight insignificant systemic toxicity *in vivo* laser-induced RVO was correlated with minimal adverse effects of the treated animals initially decreased on day 1 due to the prolonged anesthesia session and less oral intake that day. The rabbit body weight subsequently increased for 7 days post-treatment, suggesting insignificant systemic toxicity. Therefore, *in vivo* laser-induced RVO was correlated with minimal adverse effects.

**Figure 7:**
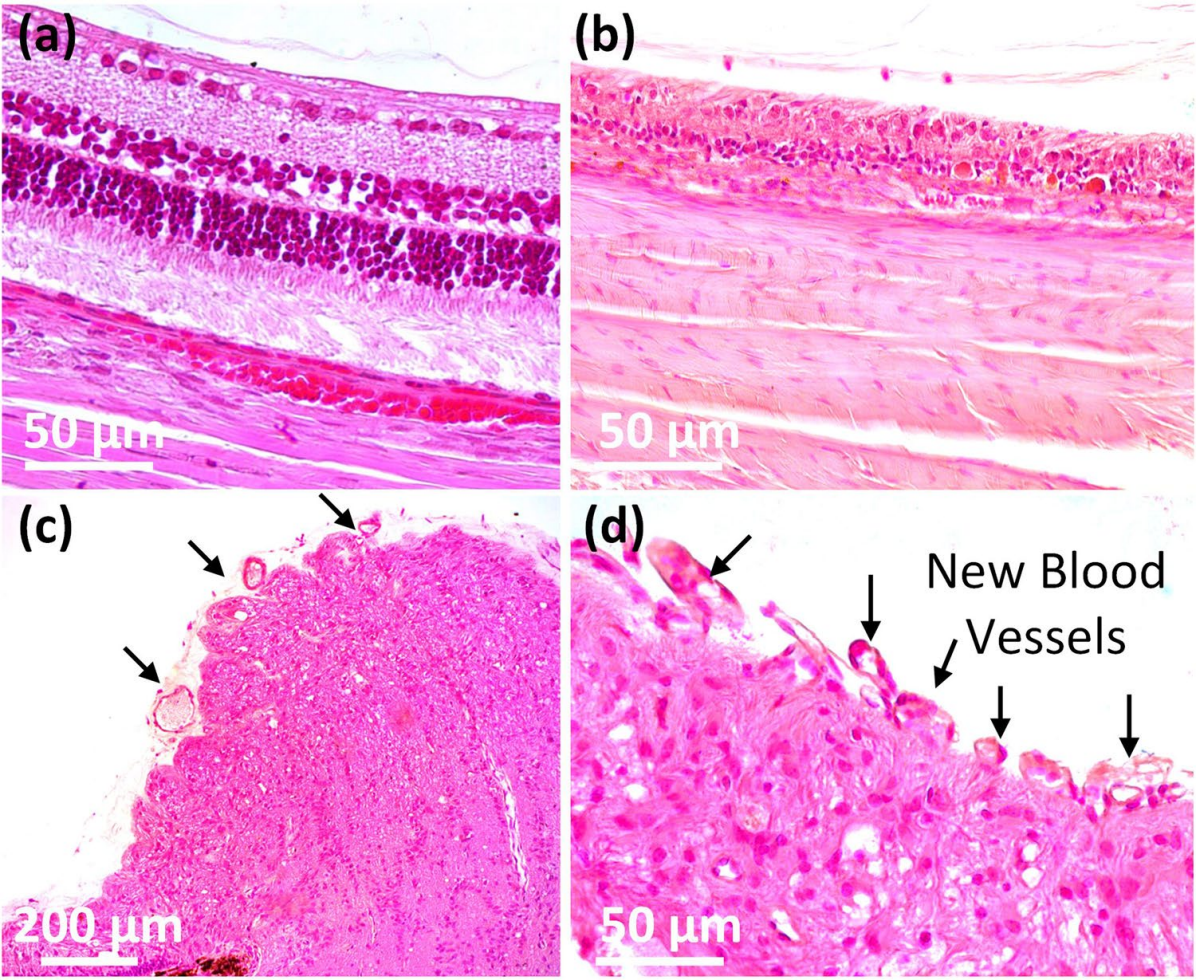
Histological analysis after laser-induced RVO in rabbit retina. H&E-stained images of RVO obtained from various groups: control (**a**), and treatment (**b**). The histological change and RNV development were observed at: (**c**) ×20 and (**d**) ×40.

To further evaluate the establishment of RNV and photothermal effect on retinal thickness, histological analysis was implemented 28 days after treatment by using a standard hematoxylin and eosin (H&E) stain as shown in Fig. 7. On the control histological images, most cellular structure and nuclei were visualized without any significant changes. The morphology of cellular structures was not changed, and most nuclei were easily observed (Fig. 7(a)). On the tissues treated with laser irradiation, the retinal thickness was significantly changed (Fig. 7(b)). Regions of the retina that were treated with laser were estimated to be 30.36 ± 2.24 thick, while control tissues were 124.70 ± 3.44 µm thick. Retinal regions impacted by laser-induced RVO were approximately 4.1-fold thinner than untreated regions. Finally, tissues extracted from with laser irradiation with the assisted of Rose Bengal showed a change in morphology. New blood vessels were easily observed as indicated by the black arrows in Fig. 7(c,d). These results indicate that photothrombotic vascular occlusion affected retinal vessels in the presence of the light-sensitive dye-Rose Bengal and indicate the potential of photocoagulation to achieve retinal vein occlusion and create RNV.

## Discussion

This is the first study to successfully demonstrate detection and visualization of RVO and RNV in the rabbit retina using a multimodal PAM and OCT imaging platform. This is important because PAM has a higher spatial resolution, comparable resolution for comprehensive anatomic and functional retinal characterization, three-dimensional capabilities, and evaluates the optical absorption changes in the retinal microvasculature at several time points to better characterize and assess the retinal vasculature. The equipment characterized the boundaries of the treated regions, compared to the adjacent tissues. As a result of its strong absorption at 532 nm, biocompatible Rose Bengal can enhance the thermal effects of laser irradiation to induce RVO without significant toxic effects. Furthermore, this is also the first study demonstrating RNV can be monitored and assessed using multimodal PAM and OCT imaging equipment *in vivo* in the rabbit. The multimodal PAM and OCT imaging system has a notable capability for resolving individual microvasculature. Using an energy which is below half of the ANSI safety limit (~80 nJ), the PAM imaging could non-invasively, and label-free, visualize single RNV vessels. The OCT system could help to observe single RNV capillaries and distinguish separate layers of retina, choroid, RNV, and sclera. Both PAM and OCT can identify the variation in retinal thickness. PAM provides depth information in a non-invasive manner, and when compared to OCT, it is more readily able to identify the location of choroidal and deep vasculature. In comparison with currently available imaging techniques, the multimodal system allows the detection and visualization of individual capillaries with high contrast and high resolution. While OCT is widely used to image retinal vasculature, retinal occlusion, and RNV models, OCT has limited imaging penetration depth^21^.

Both McAllister *et al*. and Cohofski *et al*. have presented that RVO can be monitored with FA^19,36^. However, FA imaging could not determine the 3D depth of the RVO, resulting in limitation of visualization of the blood vessel network. To overcome these limitations, this study combined OCT and PAM to enhance visualize retinal occlusion as well as RNV. The multimodal imaging equipment has a lateral and axial resolution of 4.1 and 37 µm respectively for PAM and 3.8 and 3.0 µm respectively for OCT^11^. Acquired 3D OCT and PAM allow researchers to monitor the RVO and resulting formation of RNV. PAM was able to visualize RVO with high contrast to monitor the changes in microvasculature after laser irradiation.

The PA images presented in Fig. 3(b) characterized the boundaries of the treated regions, compared to the adjacent tissues. The decreased PA amplitudes from the occluded vessels may have been a result of the change in optical absorption coefficients (µa = 270.56 cm^−1^ for oxygen hemoglobin vs. µa = 199.91 cm^−1^ for deoxygenated hemoglobin at 580 nm^41^). Importantly, PAM and OCT were able to visualize the changes in retinal vessels in both 2D and 3D. According to Fig. 6(a), the mean diameter of the treated retinal vessels was approximately 24% shorter than the retinal vessels before laser irradiation. Additionally, the high-resolution PAM system could detect single microvessels without requiring the administration of photoacoustic contrast agents, which is required in clinical applications. Yet, the challenge of the current system is that the axial resolution is approximately 37 µm, which is larger than the diameter of RNV (~10 to 50 µm)^42^, making it difficult to discern individual RNV vessels using volumetric PAM data. Thus, future efforts will focus on improving the axial resolution of the current imaging system so that microvasculature under 10 µm in diameter can be detected.

Another advantage of the current multimodal PAM and OCT system is that OCT can be used to retinal vessels in real-time. Additionally, the PAM system can acquire rapid (<1 minute), high-resolution images, which is faster than the conventional PA system which uses mechanical scanning methods. Liu *et al*. has reported a PA scanning time of approximately 20 min to achieve 3 × 3 mm^2^ image^43^. Another study reported by Hu *et al*. describe the total image acquisition time of about 2 h to achieve an image of 4 × 4 mm^2^ (scanning step size of 2.5 µm)^44^. de la Zerda *et al*. used wide-field illumination technique to acquire PA images, but the acquisition time remained about 90 min for a 12 × 8 mm^2^ area^27^. However, the acquisition speed of this current system will still need to be improved for clinical translation. The imaging speed is limited by the laser repetition rate of 1 kHz of the optical parametric oscillator (OPO), but this can readily be increased to a fraction of the current time by switching to commercially available high-speed laser source that is not tunable. For example, by using a 10 MHz nanosecond pulse duration single wavelength laser illumination, the scanning speed can be reduced to approximately 10 s. A second limitation of the current imaging system is that the field of view of PAM is limited by the ultrasound needle-shaped transducer. To acquire larger imaging regions with this system, several volumes need to be recorded at different positions and then overlaid, which is time consuming and requires excellent compliance of the post-imaging processing technique. A more efficient method can be performed in real-time by using an array of ultrasound transducers to record a larger volume image.

Creating RVO models in rodents has previously been investigated^8,21,36^. Cehofski *et al*. have shown that a rabbit RVO model can be created through concurrent application of 250 mW laser and Rose Bengal^36^. Additionally, this same study demonstrated that the retinal vein remained unaffected when the retinal vessels were illuminated with 250 mW laser without Rose Bengal, indicating that Rose Bengal may enhance the absorption of laser light and reduce the required laser energy. Additionally, these studies have reported that there was no RNV observed in the animal eyes after laser irradiation. In contrast, Udin *et al*. demonstrated that RNV was observed in the eyes of murine mice 10 to 14 days after the development of a RVO^8^. Similarly, RNV was observed in albino rats at 2 weeks and pigmented rats at 10 to 12 weeks. Yet, mice and rat eyes are much smaller than human eyes (i.e., ~3 mm for mice, ~6 mm for rats vs. ~18 mm for rabbits and ~23 mm for human).

The present study found that RNV developed in rabbit models 28 days after photocoagulation, and the RNV was stable from day 28 to day 90. As shown in Figs 4 and 5, RNV formed at two locations: at the laser irradiation site and around the optic disc, Fig. 4(b), and at the optic nerve only when the retinal vessels were completely eradicated (Fig. 5(b)). Interestingly, the RNV was stable for up to 2 months (Fig. 4). However, the limitation of our study is that RVO was created using high laser power irradiation (150~300 mW) and multiple shots (~20 shots for each treatment) which may cause damage the retina, RPE, and choroid. Fewer laser spots may be an advantage to create venous thrombus without significant damage to the retinal vein^36^.

Another challenge is that this technique was developed to mimic human RVO. However, photothrombotic induced RVO differs from human RVO. In photothrombotic induced RVO, thrombosis simultaneously occurs within several vessels in the irradiated site, whereas human RVO related ischemia is typically a result of interrupted blood flow within a single artery or vein. Local interruption of blood flow is initially less harmful to adjacent vessels as these vessels may receive blood supply by collateral arteries and do not undergo necrotic cell death. To reduce these side effects, future studies will focus on developing an integrated image-guided laser source for inducing RVO. The laser spot size can then be controlled, and the RVO model can be monitored in real-time by achieving high-resolution and 3D using photoacoustic imaging, and high sensitivity, high specificity, and high-resolution cross-sectional imaging of RNV using OCT imaging.

In summary, this is the first study to demonstrate that RNV can be observed in the rabbit Rose Bengal RVO model using multimodal fundus photography, FA, OCT, and PAM imaging. As a result of its strong absorption at 532 nm, biocompatible Rose Bengal can enhance the thermal effects of laser irradiation to induce RVO without significant toxic effects. Furthermore, our results indicate that RNV can remain stable for up to 2 months. This is also the first study demonstrating RNV can be monitored and assessed using multimodal PAM and OCT imaging equipment *in vivo* in the rabbit. The multimodal PAM and OCT imaging system has a notable capability for resolving individual microvascular. Using an energy which is below half of the ANSI safety limit (~80 nJ), the PAM imaging could non-invasively, and label free image single RNV vessels. The OCT system could help to observe single RNV capillaries and distinguish separate layers of retina, choroid, RNV, and sclera. In addition, both PAM and OCT can identify the variation in retinal thickness. Therefore, the multimodal ocular imaging system can be effective and safe for visualization and characterization of RNV.

## Materials and Methods

### Chemical materials

All chemicals and reagents were purchased from the approved vender and directly used as received without further purification. Rose Bengal was purchased from Sigma-Aldrich (Sigma, St. Louis, Mo, USA). Phosphate-buffered saline (PBS) was obtained from Gibco (BRL, Life Technologies, Grand Island, NY, USA). Indocyanine green (ICG) and 10% fluorescein sodium was purchased from Akorn (Akorn, Lake Forest, IL, USA). Xylazine was ordered from MWI Animal Health (Anased®Boise, ID, USA). Ketamine was acquired from JHP Pharmaceuticals (JHP Pharmaceuticals, Rochester, MI, USA).

### Animal model preparation

All animal experimental protocols were approved by the Institutional Animal Care and Use Committee (IACUC) of the University of Michigan (Protocol number: PRO00008566 and PRO00006486, PI: Y. Paulus) and performed in accordance with the Association for Research in Vision and Ophthalmology (ARVO) Statement on the Use of Laboratory Animals in Ophthalmic and Vision Research.

Eight New Zealand rabbits (both genders) with the age of 2–4 months and weighing of 1.9–3.0 kg were received from the Center for Advanced Models and Translational Sciences and Therapeutics (CAMTrasST) at the University of Michigan Medical School. All of the animals received retinal vein occlusion by laser photocoagulation. The rabbits were intravenously injected of 2.2 mL Rose Bengal (50 mg/mL) dispersed in phosphate-buffered saline (PBS) at 50 mg/kg, and were treated with laser irradiation described below (N = 8). Prior to the experiments, the animal vial such as body heat, heart rate, mucous membrane color, and respiratory rate were supervised using a pulse oximeter (V8400D Capnograph & SpO2 Digital Pulse Oximetry, Smiths Medical, MN, USA). A mixed solution of ketamine (40 mg/kg IM, 100 mg/mL) and xylazine (5 mg/kg IM, 100 mg/mL) were then intramuscularly (IM) injected into each rabbit model for anesthesia. The pupils of the rabbit were dilated with tropicamide 1% ophthalmic and phenylephrine hydrochloride 2.5% ophthalmic. In addition, 0.5% Topical tetracaine was instilled in the eye for topical anesthesia. Few drops of lubricant (Systane, Alcon Inc., TX, USA) was added into the eye every minute to avoid corneal dehydration. A vaporized isoflurane anesthetic (1 L/min oxygen and 0.75% isoflurane) (Surgivet, MN, USA) was supplied to maintain anesthesia during the experiments.

### Retinal vein occlusion (RVO)

To create the retinal vein occlusion in the retinal veins, dye-enhanced photothrombosis was performed as defined previously by Oncel *et al*. and Nguyen *et al*.^45,46^. In brief, a contact lens (Volk H-R Wide Field, laser spot 2x magnification, Volk Optical Inc, Mentor, OH, USA) coupled with the cornea of the rabbit eye using 2.5% Gonak Hypromellose Ophthalmic Demulcent Solution (Akorn, Lake Forest, IL, USA). The target veins were observed and defined from the slit lamp. Then, Rose Bengal with concentration of 50 mg/mL was intravenously injected into the rabbit. 5–10 seconds after the injection, a 532-nm green laser light (power = 150–300 mW, spot size = 75 µm, and the irradiation time = 0.5 s per spot) was used to shined into the rabbit eye (Vitra 532 nm, Quantel Medical, Cournon d’Auvergne, France). Each the retinal vein received twenty shots of the laser illuminations (150 mW) at the same position until the blood vessel was completely occluded and the blood flow was stopped. In addition, 20 shots of laser light was further applied at the power of 300 mW to inhibit reopening of the vein^38^.

### RVO monitoring

Fifteen minutes after the laser treatment, the rabbits’ retinal vasculature were monitored with a multimodal imaging system including digital color fundus photography, fluorescein angiography (FA), and integrated Optical Coherence Tomography (OCT) with photoacoustic microscopy (PAM). In addition, the rabbit model was further examined and evaluated with the multimodal imaging equipment on different time points: days 4, 28, 35, 49 and day 90 after laser-induced RVO as shown in Fig. S5.

### Color fundus

All retinal veins pre and post laser illumination were imaged using 50 degree color fundus photography (Topcon 50EX, Topcon Corporation, Tokyo, Japan) to select the target vessels for laser irradiation and to monitor the position of photothrombotic after treatment. In addition, the color fundus images were also used to measure the proportion of blood perfusion after laser irradiation. To monitor the effect of the laser to adjacent vessels, color fundus images were acquired at different positions of the eye such as the optic nerve, the superior retina above the optic disc, the inferior retina below the optic disc, the temporal medullary ray, and the nasal medullary ray.

### Fluorescein angiography (FA)

Fluorescein angiography (FA) was simultaneously acquired after taking color fundus photographs to evaluate the vasculature and confirm vascular occlusion. The Topcon 50EX system was used to obtained FA images as described by previous studies^11^ [Add BEO paper,Phuc]. A sodium fluorescein solution at a concentration of 10% fluorescein was injected in the marginal ear vein at dose of 0.2 mL (Akorn, Lake Forest, IL, USA). Immediately after injection, the FA images were subsequently acquired and then late phase FA images were acquired at every minute for a period of at least 20 minutes.

### Dual modality imaging equipment

**Figure 8:**
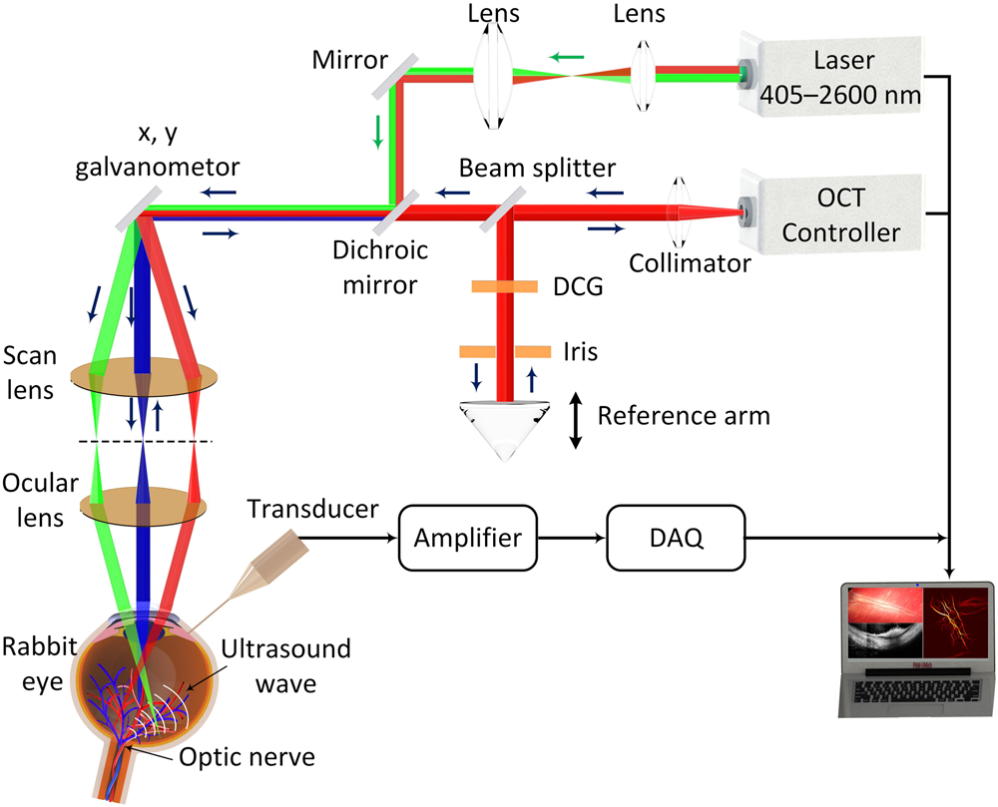
Experimental setup of the multimodal molecular ocular system with combined photoacoustic microscopy (PAM) and optical coherence tomography (OCT). DCG: dispersion compensation glass.

A custom-made multimodal PAM and OCT system (Fig. 8) was employed as previously described^11^. For PAM, a short laser pulsed (pulsewidth = 3~5 ns and pulse repetition rate = 1 kHz) generated by diode-pumped solid-state laser (NT-242, Ekspla, Vilnius, Lithuania, tunable wavelength range 405–2600 nm) was used to illuminate into the tissues. The laser light was then filtered, collimated and spread to an optical galvanometer. The laser beam delivered to a scan lens (OCT-LK3-BB, Thorlabs, Inc., Newton, NJ) and collimated with an ophthalmic lens (OL, AC080–010-B-ML, Thorlabs) to create a parallel circular laser spot with an estimated diameter of 2 mm. The laser beam was finally focused on the fundus by the rabbit eye optics with an estimated diameter of 20 μm. The average laser light energy on the eye used to induce photoacoustic signal was estimated to be 80 nJ at 580 nm, which is half of the American National Standards Institute limit of the maximum permissible single laser pulse energy on the retina^11^. A 27.0 MHz custom-built ultrasonic transducer (two-way -6dB bandwidth 60% (Optosonic Inc., Arcadia, CA, USA) was applied to acquire the photoacoustic signals. The lateral and axial resolutions of the PAM system were first calibrated at the focal plane of the scan lens and estimated to be 4.1 and 37.0 µm, respectively by using the full-width at half-maximum (FWHM).

The PAM images were carried out at an optical wavelength of 580 nm, which matches the optical absorption peak of oxyhemoglobin. The analogue PA signals were amplified using a low-noise amplifier (gain 57 dB, AU-1647, L3 Narda-MITEQ, NY) and converted into digital signals using a high-speed digitizer at a sampling rate of 200 MHz (PX1500-4, Signatec Inc., Newport Beach, CA). The PAM images were reconstructed by combining a sequences A-scans along the along x- and y-directions using an optical-scanning galvanometer. For a 1.5 × 1.5 mm^2^ field of view with a resolution of 6.1 × 6.1 µm^2^, the acquisition time was about 60 s. Additionally, to visualize the margin of the blood vessel, 3D image reconstruction and semi-automatically image segmentation were performed to distinguish the locations of retinal veins, retinal arteries, choroidal vasculature, and neovascularization using Amira software (FEI, Hillsboro, OR). FA images were used to identified the major retinal arteries and veins. Delay in the presence of the fluorescein dye into the central retinal vascular indicates the presence of a retinal vein occlusion (see Fig. S1).

Optical Coherence Tomography (OCT) was developed from a Thorlabs OCT system (Ganymede-II-HR, Thorlabs, Newton, NJ) by modifying the optical components^11^. In brief, the ocular lens was added into the system after the scan lens, and a dispersion compensation glass was added in the reference arm. The objects were excited by two super luminescent diodes with center wavelengths of 846 nm and 932 nm. The lateral and axial resolutions are approximately 3.8 µm and 4.0 µm, respectively. The OCT light spread through the optics system, delivered to the scanning head (shared with PAM) and was coaxially aligned with the PAM system. High-speed and high-resolution OCT images were obtained with the A-line acquisition rate of 36 kHz. The acquisition time is approximately 0.103 seconds where 512 × 1024 A-lines were recorded per image.

### *In vivo* PAM/OCT for retinal vein occlusion

Laser-induced retinal vein occlusion (RVO) models were monitored by integrated PAM and OCT modalities. Pre and post laser induced retinal ischemia and RVO, the retinal vessels were visualized using PAM and OCT equipment. PAM detects the absorption of photons by major chromophores, such as oxyhemoglobin and deoxyhemoglobin in blood. In contrast, OCT detects backscattered photons by low-coherence interferometry. Matlab user interface (MathWorks, Massachusetts, USA) was developed to control the imaging systems. For *in vivo* imaging, the rabbit’s head and body were positioned on two different stabilization platforms to avoid motion artifacts. A heat blanket (TP-700, Stryker Corporation, Kalamazoo, MI) were applied to maintain the body temperature of the rabbit during the experiment. The region of interest (ROI) were selected and imaged with color fundus photography, FA and OCT. Afterwards, an ultrasonic transducer was placed in the eye chamber to acquire the baseline PAM images. Simultaneously, Rose Bengal-induced RVO was implemented on the rabbit and the vascular occlusion were imaged with PAM, OCT, fundus photography, and FA at 15 mins, day 4, 28, 35, 49, and 90 after RVO to evaluate the status of the retinal vascular (i.e., non-perfusion, re-perfusion, occluded, presence of retinal neovascularization, size of retinal vessels, and stability of RNV). After the *in vivo* experiments, the rabbit was sent back to the animal facility and the body weight was monitored for a period of 7 days.

### Histological analysis

Rabbits were sacrificed twenty-seven days after laser illumination for histological analysis to examine the variation in retinal vessels. Rabbits were euthanized by intravenous injection of euthanasia solution of 0.22 mg/kg I.V at concentration of 50 mg/ml (Beuthanasia-D Special, Intervet Inc., Madison, NJ, USA). All of their eyes (treated eyes and untreated eyes), and other tissues (heart, lung, spleen, liver, and kidney) were collected from the euthanized rabbits for histological analysis. The harvested organs were fixed overnight in 10% neutral buffered formalin (VWR, Radnor, PA, USA). Davidson’s fixative solution (Electron Microscope Sciences, PA, USA) was used to fix the collected eyeball for 8 hours and kept at room temperature to avoid retinal detachment. The semi-processed eyeball was then shifted to 50% of alcohol solution (Fisher Scientific, PA, USA) for a minimum of 24 hours. Lastly, the tissue was moved to 70% alcohol solution and kept at room temperature for further 24 h. The fixed tissues were embedded in paraffin and cross-sectionally cut into a thickness of 4 µm using a Leica autostainer XL (Leica Biosystems, Nussloch, Germany). Subsequently, the sectioned tissues were stained with hematoxylin and eosin (H&E) for standard histopathological examination. To detect the position of retinal neovascularization, the H&E images were observed under Leica DM6000 microscope and digital images were capture using the BF450C camera (DM600, Leica Biosystems, Nussloch, Germany).

### Statistical analysis

All experiments were carried out a minimum of four times. Quantification of the photoacoustic (PA) amplitudes from each retinal vessel were calculated and the final data are presented as the mean ± standard deviation (SD). Regions of interest (ROI), approximately 30 pixels, were chosen at the same field of view with each vessel to calculate the PA amplitudes and standard deviations. One tail student’s t-tests were performed at each time point independently to compare two experimental conditions. and *P* ≤ 0.05 was considered as statistically significant (as shown in Dataset).

## Supplementary information

Supplementary Information

Visualization 1

Visualization 2

Dataset

## References

[1] Wong TY, Scott IU (2010). Retinal-vein occlusion. N Engl J Med.

[2] Hayreh SS (2014). Ocular vascular occlusive disorders: natural history of visual outcome. Prog Retin Ey Res.

[3] Rogers S (2010). The Prevalence of Retinal Vein Occlusion: Pooled Data from Population Studies from the United States, Europe, Asia, and Australia. Ophthalmology.

[4] Jonas, J., Paques, M., Monés, J. & Glacet-Bernard, A. In *Macular Edema* Vol. 47 111–135 (Karger Publishers, 2010). 10.1159/000320076 20703046

[5] Rehak M, Wiedemann P (2010). Retinal vein thrombosis: pathogenesis and management. J. Throm. Haemost..

[6] Li Jia, Paulus Yannis M., Shuai Yuanlu, Fang Wangyi, Liu Qinghuai, Yuan Songtao (2017). New Developments in the Classification, Pathogenesis, Risk Factors, Natural History, and Treatment of Branch Retinal Vein Occlusion. Journal of Ophthalmology.

[7] Hayreh SS (1983). Classification of central retinal vein occlusion. Ophthalmology.

[8] Uddin MI, Jayagopal A, McCollum GW, Yang R, Penn JS (2017). *In vivo* imaging of retinal hypoxia using hypox-4-dependent fluorescence in a mouse model of laser-induced retinal vein occlusion (RVO). Invest Opthalmol Vis Sci.

[9] Nguyen VP (2017). Doxorubicin-fucoidan-gold nanoparticles composite for dualchemo-photothermal treatment on eye tumors. Oncotarget.

[10] Nguyen VP (2017). Biocompatible astaxanthin as a novel marine-oriented agent for dual chemo-photothermal therapy. PloS one.

[11] Tian C, Zhang W, Mordovanakis A, Wang X, Paulus YM (2017). Noninvasive chorioretinal imaging in living rabbits using integrated photoacoustic microscopy and optical coherence tomography. Opt Epxress.

[12] Kuehlewein L, An L, Durbin MK, Sadda SR (2015). Imaging areas of retinal nonperfusion in ischemic branch retinal vein occlusion with swept-source OCT microangiography. Ophthalmic Surgery, Lasers and Imaging Retina.

[13] Gradle HS, Meyer SJ (1929). The blind spot. *The Australasian*. Journal of Optometry.

[14] Straatsma BR, Foos RY, Heckenlively JR, Taylor GN (1981). Myelinated retinal nerve fibers. Am J Ophthalmol.

[15] Kodama T, Hayasaka S, Setogawa T (1990). Myelinated retinal nerve fibers: prevalence, location and effect on visual acuity. Ophthalmologica.

[16] Tarabishy AB, Alexandrou TJ, Traboulsi EI (2007). Syndrome of myelinated retinal nerve fibers, myopia, and amblyopia: a review. Survey of ophthalmology.

[17] Coscas F (2016). Optical Coherence Tomography Angiography in Retinal Vein Occlusion: Evaluation of Superficial and Deep Capillary Plexa. Am J Ophthalmol.

[18] Prasad PS, Oliver SC, Coffee RE, Hubschman J-P, Schwartz SD (2010). Ultra wide-field angiographic characteristics of branch retinal and hemicentral retinal vein occlusion. Ophthalmology.

[19] McAllister IL, Yu D-Y, Vijayasekaran S, Barry C, Constable I (1992). Induced chorioretinal venous anastomosis in experimental retinal branch vein occlusion. Br J Ophthalmol.

[20] Lopez-Saez M (1998). Fluorescein-induced allergic reaction. Ann Alergy Asthma Immunol.

[21] Soetikno BT (2017). Optical coherence tomography angiography of retinal vascular occlusions produced by imaging-guided laser photocoagulation. Biomed Opt Expess.

[22] Li Y, Paulus XX, Novel YM (2017). Retinal Imaging Technologies. Int. J Opthalmol Eye Scien.

[23] Roorda A (2002). Adaptive optics scanning laser ophthalmoscopy. Opt Epxress.

[24] Seeliger MW (2005). *In vivo* confocal imaging of the retina in animal models using scanning laser ophthalmoscopy. Vis Res.

[25] Spaide RF, Fujimoto JG, Waheed NK (2015). Image artifacts in optical coherence angiography. Retina (Philadelphia, Pa.).

[26] Salas M (2017). Visualization of micro-capillaries using optical coherence tomography angiography with and without adaptive optics. Biomed Opt Expess.

[27] de La Zerda A (2010). Photoacoustic ocular imaging. Opt. Lett..

[28] Hu ZWX, Liu Q, Paulus YM (2015). Photoacoustic Imaging in Ophthalmology. Int. J Opthalmol Eye Scien.

[29] Jiao S (2010). Photoacoustic ophthalmoscopy for *in vivo* retinal imaging. Opt Epxress.

[30] Silverman RH (2010). High-Resolution Photoacoustic Imaging of Ocular Tissues. Ultrasound Med Biol.

[31] Hennen SN (2015). Photoacoustic tomography imaging and estimation of oxygen saturation of hemoglobin in ocular tissue of rabbits. Exp Eye Res.

[32] Liu X (2015). Optical coherence photoacoustic microscopy for *in vivo* multimodal retinal imaging. Opt. Lett..

[33] Song, W., Wei, Q., Jiao, S. & Zhang, H. F. Integrated Photoacoustic Ophthalmoscopy and Spectral-domain Optical Coherence Tomography. *J Vis Exp*, **4390**, 10.3791/4390 (2013). 10.3791/4390 PMC3582672 23354081

[34] Tian, C., Zhang, W., Nguyen, V. P., Wang, X. & Paulus, Y. M. Novel Photoacoustic Microscopy and Optical Coherence Tomography Dual-modality Chorioretinal Imaging in Living Rabbit Eyes. *J Vis Exp* (2018). 10.3791/57135 PMC5912387 29553520

[35] Hughes A (1972). A schematic eye for the rabbit. Vis Res.

[36] Jørgensen Cehofski, L. *et al*. Dye-free porcine model of experimental branch retinal vein occlusion: a suitable approach for retinal proteomics. *Am J Ophthalmol*, **2015** (2015). 10.1155/2015/839137 PMC4433685 26064675

[37] Hart WE, Goldbaum M, Côté B, Kube P, Nelson MR (1999). Measurement and classification of retinal vascular tortuosity. International journal of medical informatics.

[38] Ameri H, Ratanapakorn T, Rao NA, Chader GJ, Humayun MS (2008). Natural course of experimental retinal vein occlusion in rabbit; arterial occlusion following venous photothrombosis. Ger J Ophthalmol.

[39] Ebneter A, Agca C, Dysli C, Zinkernagel MS (2015). Investigation of retinal morphology alterations using spectral domain optical coherence tomography in a mouse model of retinal branch and central retinal vein occlusion. PLoS One.

[40] Yue C (2013). IR-780 dye loaded tumor targeting theranostic nanoparticles for NIR imaging and photothermal therapy. Biomaterials.

[41] Prahl, S. A. http://omlc.org/spectra/hemoglobin/ A compendium of tissue optical properties (2012).

[42] Edelman JL, Castro MR (2000). Quantitative image analysis of laser-induced choroidal neovascularization in rat. Exp Eye Res.

[43] Liu W (2014). *In vivo* corneal neovascularization imaging by optical-resolution photoacoustic microscopy. Photoacoustics.

[44] Hu S, Rao B, Maslov K, Wang LV (2010). Label-free photoacoustic ophthalmic angiography. Opt. Lett..

[45] Oncel M, Peyman GA, Khoobehi B (1989). Tissue plasminogen activator in the treatment of experimental retinal vein occlusion. Retina (Philadelphia, Pa.).

[46] Nguyen VP, Li Y, Zhang W, Wang X, Paulus YM (2018). Multi-wavelength, en-face photoacoustic microscopy and optical coherence tomography imaging for early and selective detection of laser induced retinal vein occlusion. Biomed Opt Expess.

