# Author Correction: High-resolution multimodal photoacoustic microscopy and optical coherence tomography image-guided laser induced branch retinal vein occlusion in living rabbits

**DOI:** 10.1038/s41598-020-60919-1

**Published:** 2020-03-03

**Authors:** Van Phuc Nguyen, Yanxiu Li, Wei Zhang, Xueding Wang, Yannis M. Paulus

**Affiliations:** 1 Department of Ophthalmology and Visual Sciences, University of Michigan, Ann Arbor, MI 48105 USA; 2 Department of Biomedical Engineering, University of Michigan, Ann Arbor, MI 48105 USA; 3 NTT-Hi Tech Institute, Nguyen Tat Thanh University, Ho Chi Minh, Vietnam; 4 Department of Ophthalmology, Xiangya Hospital, Central South University, NO. 87 Xiangya Road, Kaifu District, Changsha, Hunan 410008 P.R. China

Correction to: *Scientific Reports* 10.1038/s41598-019-47062-2, published online 22 July 2019

This Article contains errors in the Results section where,

“It was noted that the vessel diameter of the untreated veins was (on average?) 132% higher than that of the treated veins (vessel diameter = 121.88 ± 7.18 µm for untreated veins vs. 91.88 ± 12.81 µm for treated veins; p < 0.05), indicating that the treated veins were thinner.”

should read:

“It was noted that the vessel diameter of the untreated veins was 132% higher than that of the treated veins (vessel diameter = 121.88 ± 7.18 µm for untreated veins vs. 91.88 ± 12.81 µm for treated veins; p < 0.05), indicating that the treated veins were thinner.”

“Additionally, the (average?) RNV vessel diameter was estimated to be 29.83 ± 4.17 µm, which was 97.77% thinner than choroidal vessels (59.00 ± 11.47 µm), p < 0.05.”

should read:

“Additionally, the RNV vessel diameter was estimated to be 29.83 ± 4.17 µm, which was 97.77% thinner than choroidal vessels (59.00 ± 11.47 µm), p < 0.05.”

