# Supplementary material for: High-resolution multimodal photoacoustic microscopy and optical coherence tomography image-guided laser induced branch retinal vein occlusion in living rabbits
Source: Sci Rep. 2019 Jul 22;9:10560. doi: 10.1038/s41598-019-47062-2 (PMC6646378; doi:10.1038/s41598-019-47062-2)
Supplement: Supplementary file 1 — Supplementary Information [file 41598_2019_47062_MOESM1_ESM.docx]

**High-resolution multimodal photoacoustic microscopy and optical coherence tomography image-guided laser induced branch retinal vein occlusion in living rabbits**

**Van Phuc Nguyen^1,3#^, Yanxiu Li^1#^, Wei Zhang^2^, Xueding Wang^2^, and Yannis M. Paulus^1,2* ­­­^**

^1^Department of Ophthalmology and Visual Sciences, University of Michigan, Ann Arbor, MI 48105, USA

^2^Department of Biomedical Engineering, University of Michigan, Ann Arbor, MI 48105, USA

^3^NTT-Hi Tech Institute, Nguyen Tat Thanh University, Ho Chi Minh, Vietnam

^#^ These authors contributed equally as first author.

^*^Corresponding Author:

Yannis M. Paulus, M.D., F.A.C.S.

Department of Ophthalmology and Visual Sciences

Department of Biomedical Engineering

University of Michigan

1000 Wall Street

Ann Arbor, MI 48105, USA

Email Address: ypaulus@med.umich.edu

**Supporting Information**

**Graphical Abstract**

**
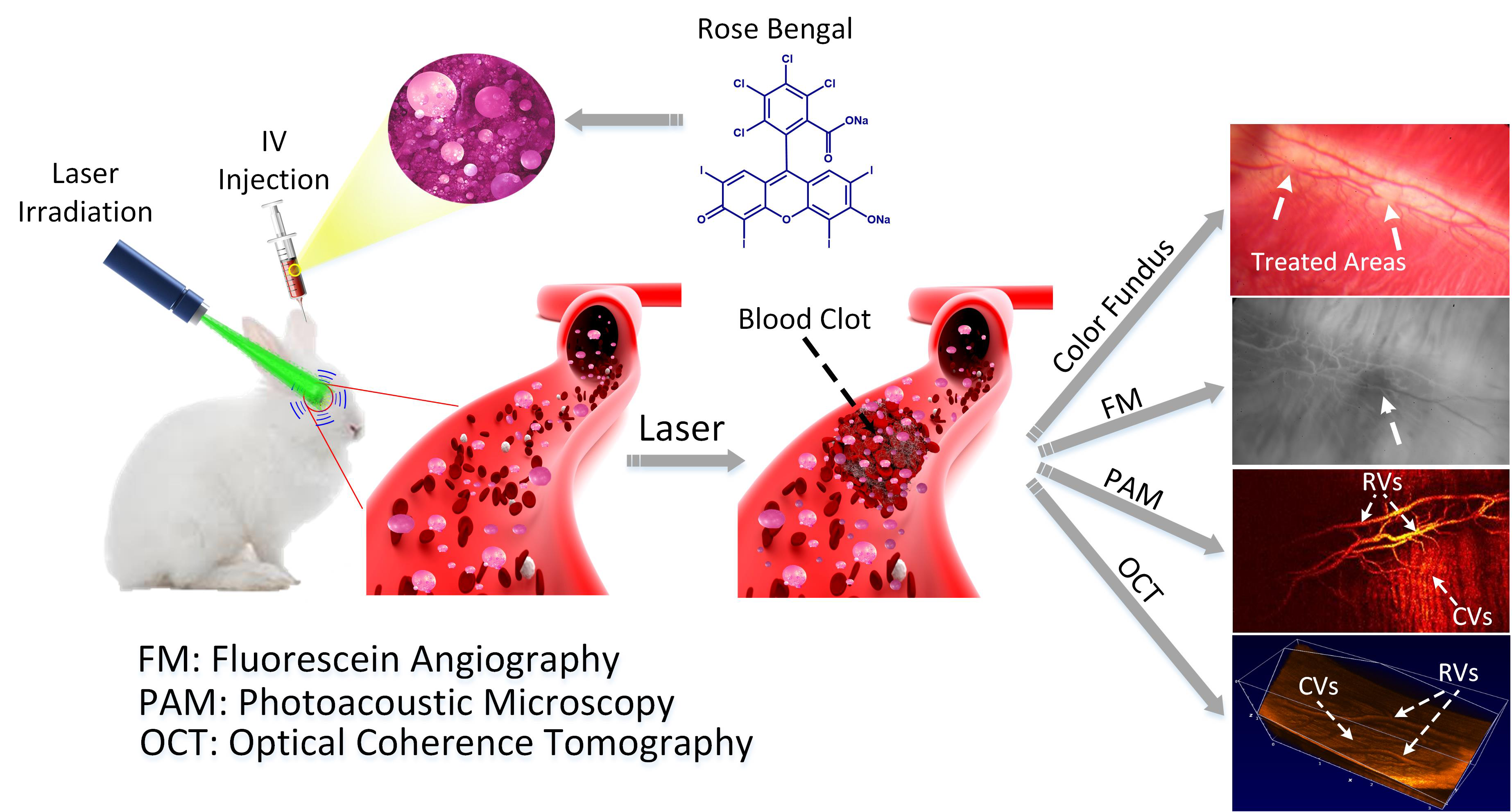
**

**
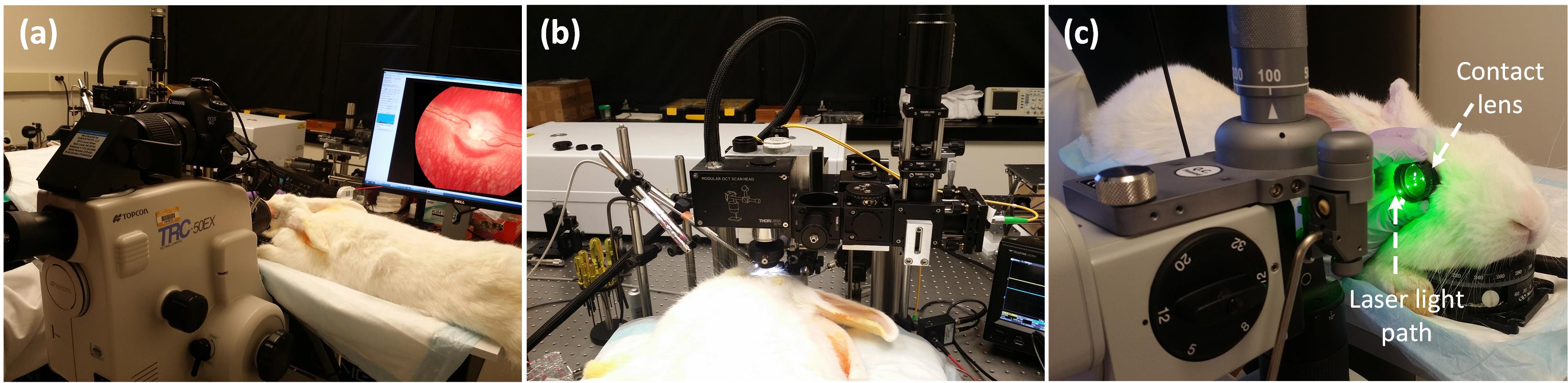
**

**Figure. S1.** Digital photograph of RVO protocol: (a) fundus color and FA system, (b) integrated PAM and OCT imaging system, and (c) Zeiss slit lamp ophthalmoscopy system. Base line retinal vessels was first acquired by fundus color/FA (a), and PAM/OCT (b). Then, RVO model was performed by using ophthalmoscopy system (c). After laser treatment, rabbit RVO model was monitored using an imaging system (a) and (b) at various times.

**
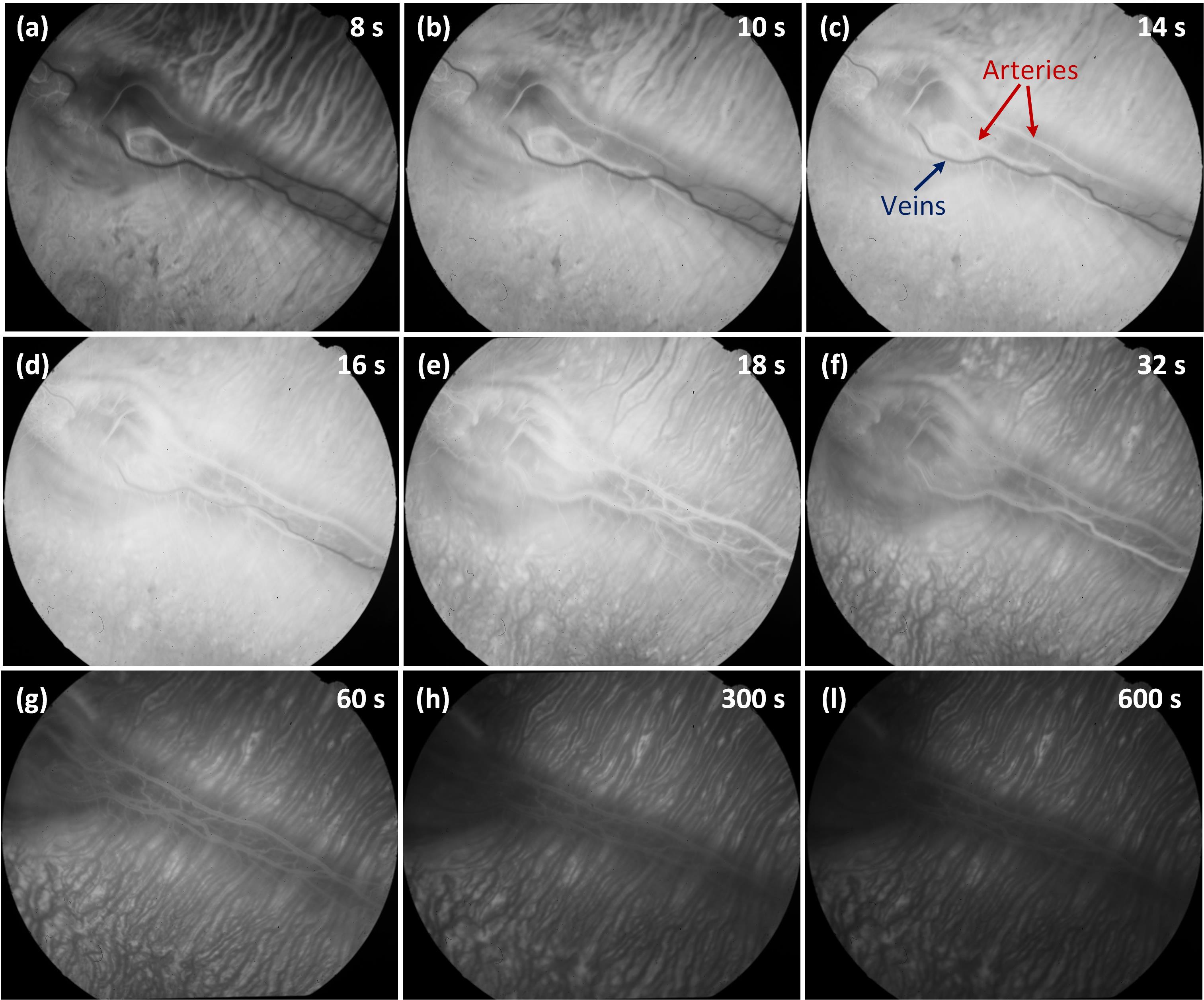
**

**Figure. S2.** Sequential fluorescein angiography images. The red arrow shows the position of arteries, whereas blue arrow depicts the location of the veins. The time on the top right indicates the number of seconds after intravenous fluorescein dye injection.


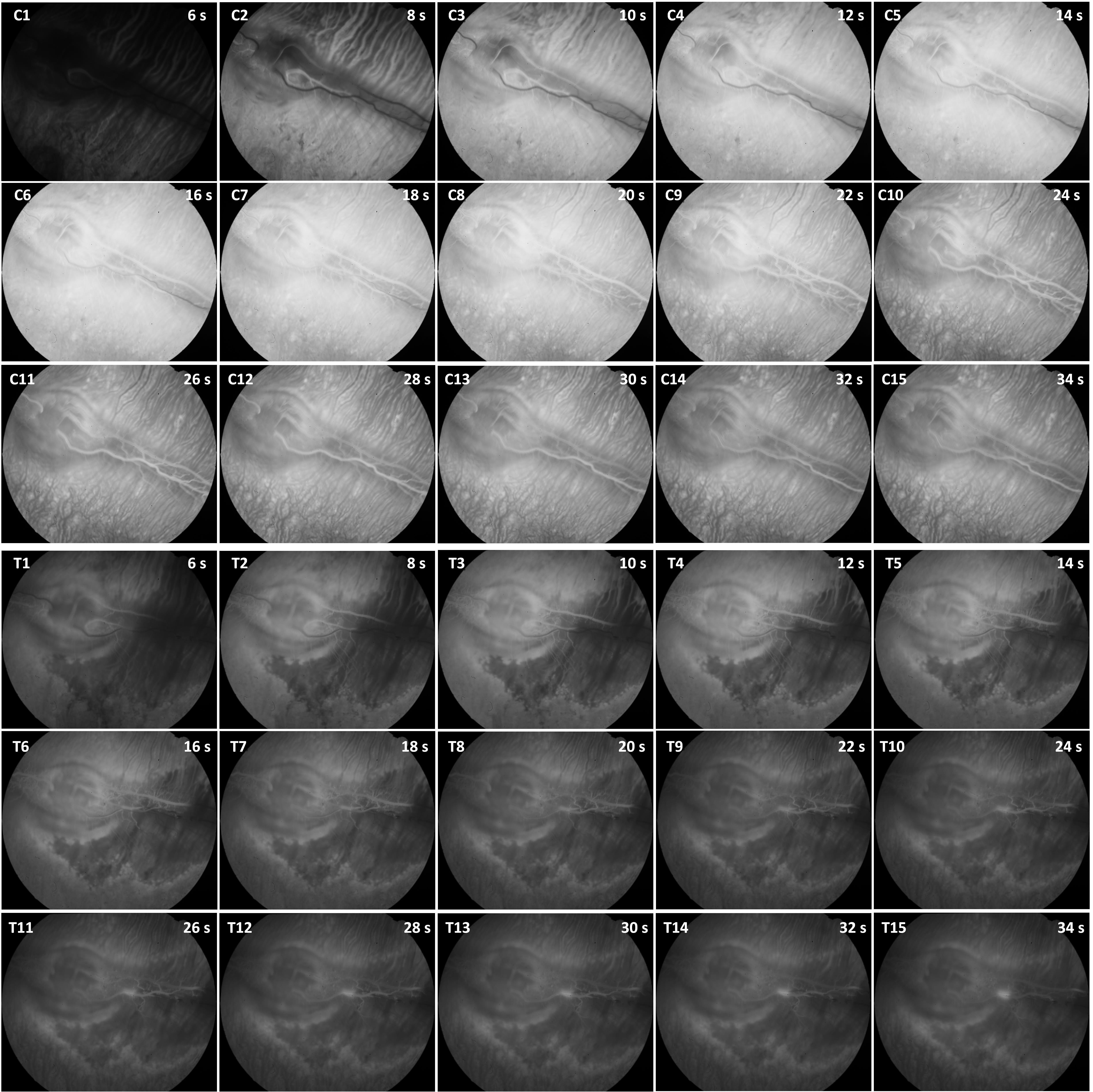


**Figure. S3**. FA images of rabbit pre- and post-laser treatment: (C1-C15) FA images of the rabbit before laser irradiation. Both artery and vein were filled with FA dyes, indicating normal blood flow without any obstacles. In contrast, the interruption of blood vessels as shown in (T1-T15), implying that blood vessels were occluded.


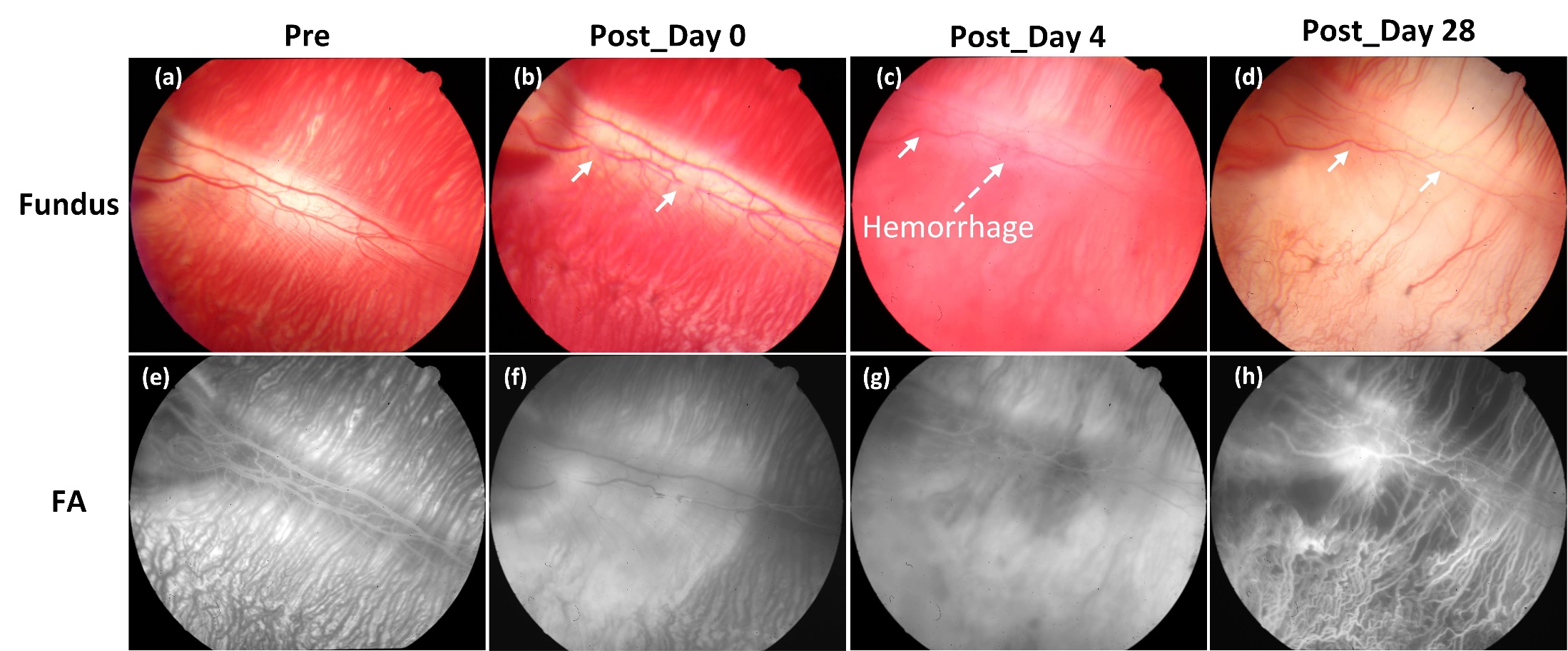


**Figure. S4**. Evaluation of retinal vessels at various treatment time. (a-d) color fundus and (e-h) FA images acquired at different treatment times (Pre, post-treatment day 0, 4 and 28, respectively). As shown in figure (c), hemorrhage appeared at the laser irradiation site. However, the hemorrhage was eradicated after treatment on day 28.

**Media:**

**Visualization 1:** 3D image reconstruction of the retinal blood vessels before laser-induced retinal vein occlusion.

**Visualization 2:** 3D volumetric rendering of the formed retinal neovascularization after laser-induced retinal vein occlusion.
